# Pyridine-Containing Macrocycles Display MMP-2/9 Inhibitory Activity and Distinct Effects on Migration and Invasion of 2D and 3D Breast Cancer Models

**DOI:** 10.3390/ijms20205109

**Published:** 2019-10-15

**Authors:** Susana Proença, Bernardo Antunes, Rita C. Guedes, Filipa Ramilo-Gomes, M. Fátima Cabral, Judite Costa, Ana S. Fernandes, Matilde Castro, Nuno G. Oliveira, Joana P. Miranda

**Affiliations:** 1 Research Institute for Medicines (iMed.ULisboa), Faculty of Pharmacy, Universidade de Lisboa, 1649-003 Lisbon, Portugal; s.proenca@uu.nl (S.P.); bernardoantunes_94@hotmail.com (B.A.); rguedes@ff.ulisboa.pt (R.C.G.); filipa.ramilo.gomes@tecnico.ulisboa.pt (F.R.-G.); fcabral@ff.ul.pt (M.F.C.); jcosta@ff.ul.pt (J.C.); mcastro@ff.ul.pt (M.C.); ngoliveira@ff.ul.pt (N.G.O.); 2 Institute for Risk Assessment Sciences, Utrecht University, P.O. Box 80177, 3508TD Utrecht, The Netherlands; 3 Centro de Química Estrutural, Instituto Superior Técnico, Universidade de Lisboa, 1049-001 Lisbon, Portugal; 4 CBIOS, University Lusófona, 1749-024 Lisbon, Portugal; ana.fernandes@ulusofona.pt

**Keywords:** pyridine-containing macrocyclic, molecular docking studies, 3D models, breast cancer, MMP inhibitors, migration and invasion

## Abstract

The role of metalloproteinases (MMPs) on the migration and invasion of cancer cells has been correlated with tumor aggressiveness, namely with the up-regulation of MMP-2 and 9. Herein, two pyridine-containing macrocyclic compounds, [15]pyN_5_ and [16]pyN_5_, were synthesized, chemically characterized and evaluated as potential MMP inhibitors for breast cancer therapy using 3D and 2D cellular models. [15]pyN_5_ and [16]pyN_5_ (5–20 µM) showed a marked inhibition of MMPs activity (100% at concentrations ≥ 7.5 μM) when compared to ARP-100, a known MMP inhibitor. The inhibitory activity of [15]pyN_5_ and [16]pyN_5_ was further supported through in silico docking studies using Goldscore and ChemPLP scoring functions. Moreover, although no significant differences were observed in the invasion studies in the presence of all MMPs inhibitors, cell migration was significantly inhibited by both pyridine-containing macrocycles at concentrations above 5 μM in 2D cells (*p* < 0.05). In spheroids, the same effect was observed, but only with [16]pyN_5_ at 20 μM and ARP-100 at 40 μM. Overall, [15]pyN_5_ and [16]pyN_5_ led to impaired breast cancer cell migration and revealed to be potential inhibitors of MMPs 2 and 9.

## 1. Introduction

Matrix metalloproteinases (MMPs) have been found to correlate with increased breast tumor aggressiveness [1,2,3,4,5]. During metastization, tumor cells recruit ECM (extracellular matrix) proteases, such as MMPs, to their leading edge for localized proteolysis of the ECM net [1,2,3,4,5]. MMPs are highly conserved proteins containing a structural Zn^2+^ ion and one to three Ca^2+^ ions, and in their catalytic domain a catalytic Zn^2+^ ion coordinated through the conserved motif HExGHxxGxxH [6]. Other common structures to MMPs are their hemopexin-like C-terminal domain essential for its non-proteolytic functions, and a pro-peptide domain which coordinates the catalytic Zn^2+^ ion maintaining MMPs latency, being its cleavage required for activation [6,7]. Although grouped according to their ECM substrate and domain organization, MMPs have a substantial substrate overlapping, being able to virtually cleave any ECM component, and in some cases, growth factors, cell adhesion molecules and other proteases precursors [8,9,10]. Development of MMPs inhibitors has thus been attempted within the framework of cancer therapy, including breast cancer [11].

**Figure 1:**
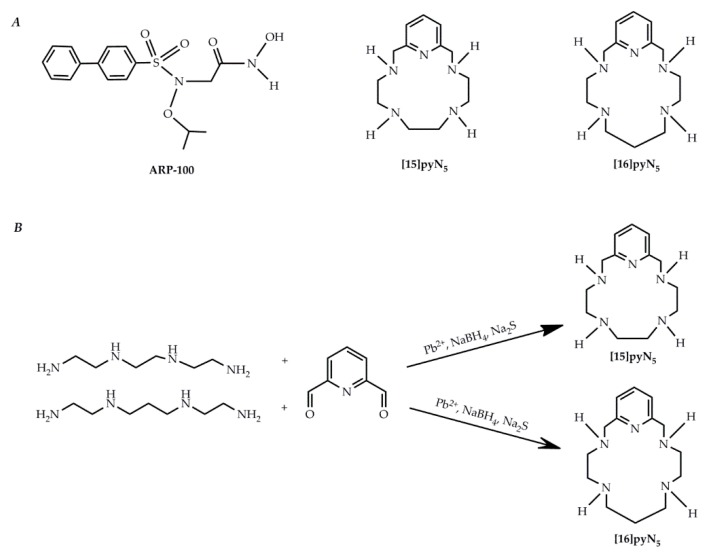
Pyridine containing macrocyclic compounds and ARP100 (**A**) chemical structures; and (**B**) schematic synthesis of [15]pyN_5_ and [16]pyN_5_.

Although initially promising, the first and second generations of MMP inhibitors were not clinically efficient due to issues related to their administration route [12] and the promotion of side effects such as the musculoskeletal syndrome (MSS) [13], respectively. Therefore, the following generation of inhibitory compounds was designed focusing on specific structures of MMP-2 and 9 gelatinases [14,15], namely giving a major emphasis to the S1′pocket, a differential structure among MMPs. A set of new molecules screened for MMP inhibitory activity were developed using the arylsulfonamide group, contained in the prinomastat, as structural model [16,17]. From this study, ARP-100 (Figure 1A) showed to be the most promising inhibitor, considering both its potency for MMP-2 inhibition and its selectivity for this MMP over MMP-1, 3 and 7. Following the hypothesis that the MSS side effects and lack of efficiency from the earlier MMPi were due to the zinc-binding group hydroxamic acid, studying alternative zinc-binding groups poses itself as a pertinent approach. Indeed, Jacobsen et al. [18] showed that aza-macrocyles have an improved MMP-3 inhibition potency when compared with one of the most common zinc binding groups, the acetohydroxamic acid (AHA). Macrocyclic compounds have also been described as potentially more specific than open chain chelators due to its ring rigidity, which constrains the type of metal that may be coordinated [19]. On the other hand, these macrocycles are much more selective, as they do not inhibit the non-heme iron enzyme soybean lipoxygenase. Two pentaaza-macrocyles containing pyridine in their backbone, [15]pyN_5_ and [16]pyN_5_ [20], synthesized (Figure 1B) and characterized by our group (Figures S1 and S2, Tables S1 and S2), display high stability constants for Zn^2+^. In a previous report we also demonstrated the protective chelating properties of [15]pyN_5_ in the context of the cytotoxicity of cadmium(II), another group 12 element [21]. Moreover, the copper(II) complex of [15]pyN_5_ was also previously studied as a superoxide dismutase mimic (SODm), modulating ROS and protecting MCF-10A (non-malignant cell line) from oxaliplatin treatment while increasing cytotoxicity of this drug in breast cancer cell line MCF-7 [22].

Additionally, it is also obvious that representative cell models and a set of adequate complementary migration and invasion endpoints are required for a better prediction of the impact of the novel gelatinase inhibitors in metastization. Three-dimensional (3D) cancer cell cultures have gained an increased interest in the field of biomedicine. A representative example are the spheroids that consist of compact cell aggregates that recapitulate in vivo cell-adhesion profile and better represent ECM-cell interactions [23]. In fact, the cell adhesion molecules β-integrins and E-cadherin have critical roles in the resistance to chemotherapeutics, and in overall malignancy [24,25,26]. Spheroids can also mimic the gradient of nutrients and oxygen that occurs in avascular tumor nodules, in some cases displaying a hypoxic region and even necrotic cores, giving rise to distinct cell populations: proliferating, quiescent and necrotic [23,25]. Cells from spheroids have shown transcriptomic differences in enzymes involved in mitochondrial metabolism, redox reactions and downregulation of a DNA mismatch repair enzyme, associated with acquired resistance to alkylating agents [27,28,29]. Moreover, spheroids respond differently than monolayers to compounds targeting epithelial-mesenchymal transition (EMT), as they present more stemness-related traits and proteins associated to metastization, hence being regarded as very suitable models for migration and invasion studies [24,26,30,31].

**Figure 2:**
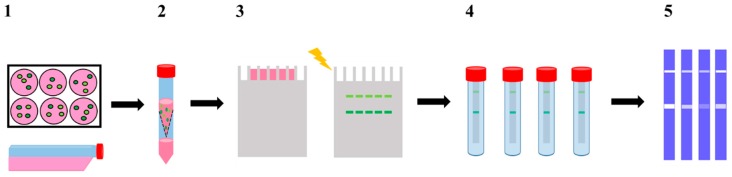
Schematic representation of the modified gelatine zymography assay. (**1**) 2D (CM2D) and 3D (CM3D) cultures conditioning in serum free media for 24 h; (**2**) CM2D/3D collection and concentration (100×); (**3**) CM2D/3D analyzed by SDS-PAGE containing 0.1% gelatine as substrate; (**4**) developing buffer incubation step of individual lanes with [15]pyN_5_, [16]pyN_5_ and ARP-100; (**5**) staining with Coomassie Brilliant Blue solution.

The work herein performed represents thus a comprehensive multidisciplinary study evaluating the effect of these pyridine containing-pentaaza macrocycles in the widely used invasive breast cancer MDA-MB-231 cells, in both 3D and 2D cultures, using different migration and invasion endpoints. The outcome of [15]pyN_5_ and [16]pyN_5_ macrocycles on MMP-2 and 9 activity was assessed by applying a modified gelatin zymography gel assay (Figure 2). To gain additional insights on the mechanism underlying the inhibitory activity, docking studies were also performed using two different scoring functions (ChemPLP and GoldScore) in order to predict protein-ligand interactions and binding modes of [15]pyN_5_ and [16]pyN_5_ on MMP-2 and MMP-9 active site.

## 2. Results

### 2.1. Viable Spheroids were Obtained from MDA-MB-231 Cells

**Figure 3:**
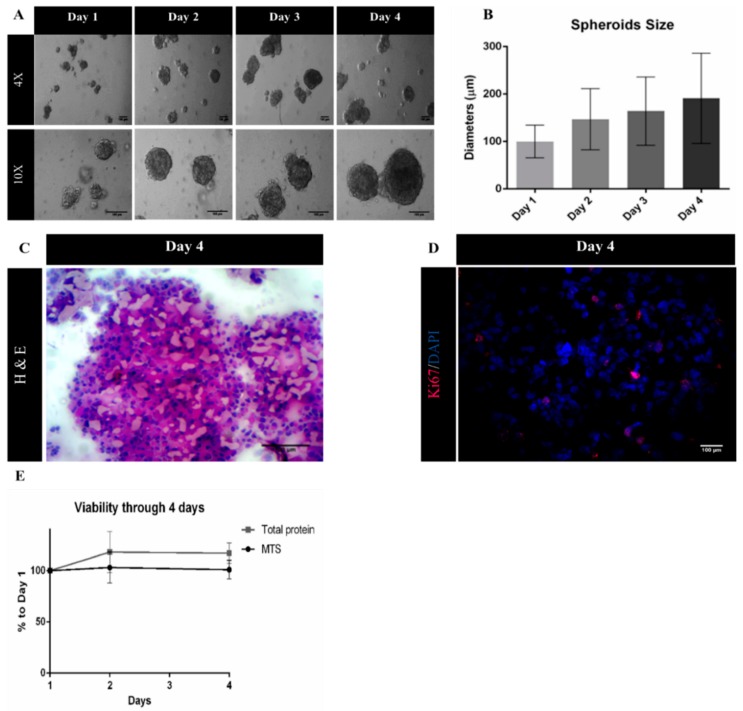
Characterization of MDA-MB-231 spheroids. **(A)** Representative phase contrast images of MDA-MB-231 spheroids from day 1 to day 4. Scale bars = 100 μm; (**B**) spheroids diameters (μm) from day 1 to day 4 represented as mean ± SD (*n* = 3); (**C**) Hematoxylin and Eosin (H&E) staining of MDA-MB-231 spheroid sections. Scale bar = 100 μm; (**D**) representative immunofluorescence images of day 4 of MDA-MB-231 spheroid cryosections labelled with Ki-67 (red). Nuclei were labelled with DAPI (blue). Scale bars = 100 μm; (**E**) cell viability/proliferation up to day 4 relative to day 1 of culture, expressed in percentage (mean ± SEM, *n* = 3).

A suspension culture-based system using ULA plates was successfully developed, as viable three-dimensional (3D) spheroids were obtained from MDA-MB-231 cells. Moreover, spheroid diameter increased during culture time, reaching a diameter of 191 µm at day 4 of culture (Figure 3A,B). At this point, the histological analysis of spheroid cryosections was performed to evaluate spheroid morphology. H&E staining of sections revealed solid spheroids, with cells homogeneously distributed and embedded in extracellular matrix (Figure 3C). Immunolabeling with proliferative marker Ki-67 (Figure 3D) showed a percentage of approximately 15% proliferating cells, which together with the MTS assay and total protein quantification data (Figure 3E) indicates a low proliferative profile of MDA-MB-231 cells when cultured under 3D cultures, a pattern that has been previously reported in the literature [32,33].

### 2.2. [15]pyN_5_, [16]pyN_5_ and ARP-100 Present a Differential Cytotoxic Profile in 2D and 3D Cultures of MDA-MB-231

**Figure 4:**
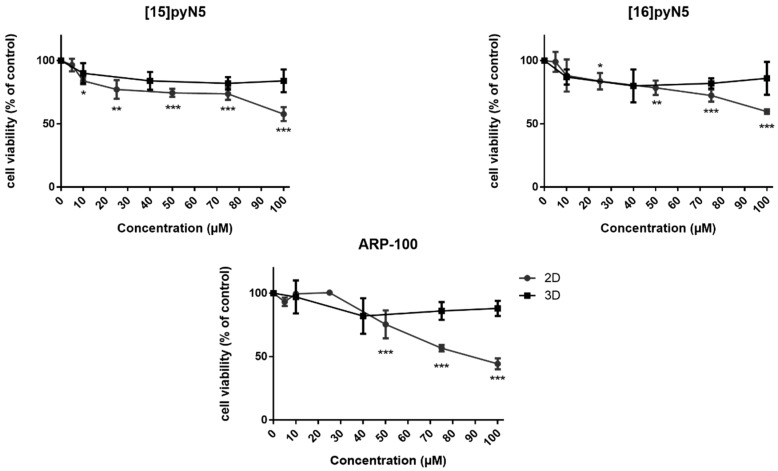
Effect of [15]pyN_5_, [16]pyN_5_ and ARP-100 on MDA-MB-231 cell viability/proliferation on 2D and 3D models. Data expressed as percentage (mean ± SD, *n* = 3–4) relative to respective controls (non-treated condition for [15]pyN_5_ and [16]pyN_5_ and 0.25% DMSO for ARP-100). Statistical significance is represented as * *p* < 0.05, ** *p* < 0.01, *** *p* < 0.001.

To evaluate the cytotoxicity of the macrocycles [15]pyN_5_ and [16]pyN_5_, dose-response curves were performed for MDA-MB-231 in both 2D and 3D cultures. The commercially available compound ARP-100 was also studied in the same conditions as a control (Figure 4). Given that the migration assays are performed in serum free conditions, the same conditions were adopted for the cytotoxicity assays. ARP-100 concentrations within the range of 1–100 μM were chosen according to the literature [34,35], being the same concentration used for [15]pyN_5_ and [16]pyN_5_ compounds.

As shown in Figure 4, in 2D, despite the cytotoxicity observed, cell viability was higher than 74% (*p* < 0.001) for concentrations up to 75 μM, decreasing to ~60% at 100 μM concentrations (*p* < 0.001) for both macrocyclic compounds. Concerning the 3D models, the cytotoxicity profile of the compounds was less pronounced, with cell viability always above 80% (n.s.). ARP-100 showed no cytotoxic effects in monolayer cultures for concentrations up to 25 μM (Figure 4). At a concentration of 50 μM, cell viability was 75% (*p* < 0.001), decreasing afterwards in a concentration dependent manner, showing a viability of 44% at 100 μM concentration of the compound (*p* < 0.001) (Figure 4).

### 2.3. MDA-MB-231 Reveal a Culture-Dependent MMP-2/9 Secretion Profile

MMPs are secreted by the cells. As such, the gelatinolytic activity of MMP-2/9 was analyzed by gelatin zymography in MDA-MB-231 conditioned medium (CM) obtained from both two-dimensional monolayer (CM2D) and three-dimensional spheroid cultures (CM3D).

Firstly, relevant parameters such as the conditioning time (24, 48, 72 or 96 h), CM concentration and quantity of total protein of CM to be loaded in the gels were optimized. Moreover, culture volume was adjusted accordingly, in order to obtain a conditioning volume per cell in 3D cultures as in the two-dimensional system. The adopted ratio was of ~200,000 cells/mL. As conditioning periods longer than 24 h in serum free conditions resulted in increased cell death (30% cell death with 0.25% DMSO in a 48-h incubation period - data not shown), a conditioning incubation period not exceeding 24 h was established. Furthermore, a volume concentration of ~100× was adopted to ensure sufficient gelatinase concentration.

**Figure 5:**
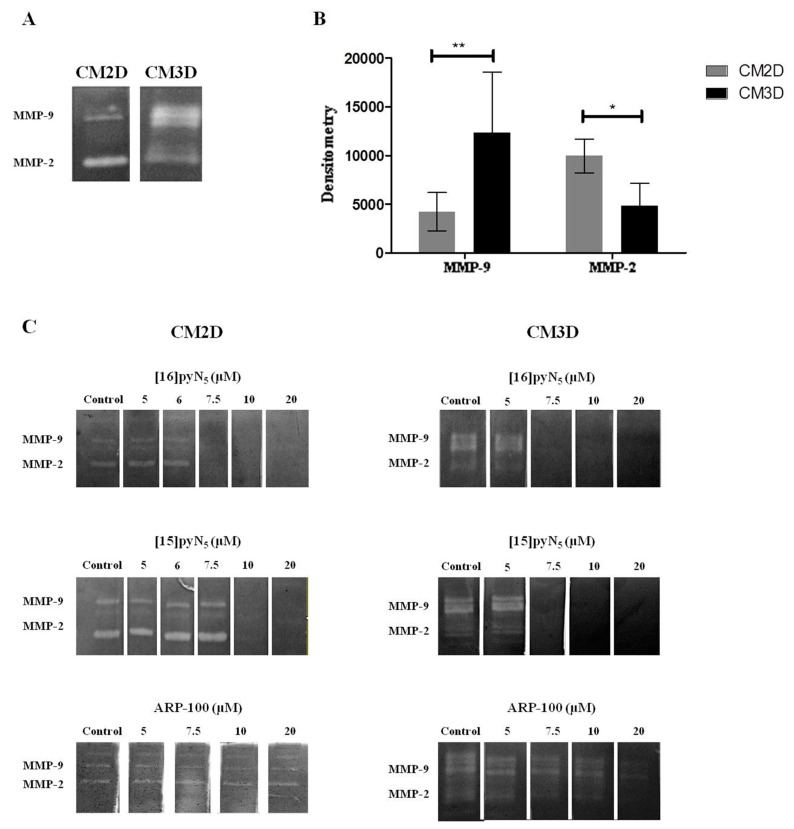
Gelatinase zymography assay. (**A**) Gel zymogram depicting differences in MMP-2 and MMP-9 content in CM2D and CM3D; (**B**) densitometric quantification of MMP-2 and MMP-9 gelatinolytic activity of CM2D and CM3D. Data expressed as mean ± SD (*n* = 3–4). Statistical significance is represented as * *p* < 0.05 and ** *p* < 0.01; (**C**) representative zymograms of CM2D/3D incubated with 5-20 µM of [16]pyN_5_, [15]pyN_5_ and ARP-100 in the developing buffer.

CM3D and CM2D zymography profiles showed a consistent differential MMP-2 and 9 activity, being MMP-2 and MMP-9 activities two-fold higher in CM2D and CM3D, respectively (Figure 5A,B). Importantly, the inhibition with EDTA (7.8 mM) confirmed that the MMP-2 (the band at 72 kDa and 66 kDa correspond to the pro- and active-form, respectively) and MMP-9 (the band at 92 kDa and 83 kDa correspond to the pro- and active-form, respectively) corresponding bands were indeed MMPs (Figure S3).

### 2.4. MMP Gelatinase Activity is Inhibited by the Macrocycles [15]pyN_5_ and [16]pyN_5_

SDS has been pointed out as a disrupting agent of TIMP-MMP binding in zymography assays, rendering in these cases the observed gelatinolytic activity independent of the TIMP (tissue inhibitors of matrix metalloproteinase) content [36]. To ensure the tested compounds would not be released from the proteins in the SDS-PAGE gel [37], as may occur for TIMPs, modifications were introduced in the zymography technique (modified zymography—Figure 2).

As such, the technique herein adopted differs from the classical zymography particularly in the developing buffer incubation step, as the compounds were dissolved in the developing buffer and then incubated with the gels containing the CM (modified zymography—Figure 2); whereas in the classical zymography the compounds are incubated directly with cells.

In our modified protocol, changes in band intensity reveal the direct effect of [15]pyN_5_, [16]pyN_5_ and ARP-100 in the MMP-2/9 gelatinolytic activity in CM2D and CM3D (Figure 5C). The macrocyclic compounds showed a more potent gelatinase inhibitory effect than ARP-100. For [16]pyN_5_ a 100% inhibition of MMP-2/9 was observed for concentrations above 7.5 μM (Figure 5C) in both CM2D and CM3D. Similarly, a 100% inhibitory effect for MMP-2/9 was observed with [15]pyN_5_ for concentrations above 7.5 μM and 10 μM in CM3D and CM2D, respectively (Figure 5C); whereas complete inhibition was not achieved with ARP-100 at any concentration studied. Importantly, a higher inhibitory effect of ARP-100 was observed for MMP-2 reflecting the specificity of ARP-100 for MMP-2.

**Figure 6:**
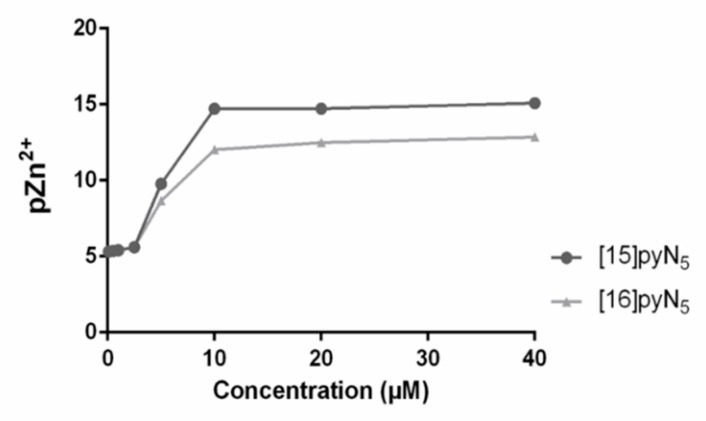
Values of pZn^2+^ (-log[Zn^2+^]), calculated for an aqueous solution of pH 7.4 containing Zn^2+^(5 μM) and [16]pyN_5_ and [15]pyN_5_ (0.1–40 µM).

In addition, the values of pZn^2+^ vs. the concentration of [15]pyN_5_ and [16]pyN_5_, were simulated resorting to the Hyss program (Figure 6) [38], using the compounds stability constants for Zn^2+^ and species distribution. The equivalence point obtained in the simulation matched the steep obtained in the zymography assay, suggesting a correlation between the chelation of free Zn^2+^ and the inhibition of gelatinases by the compounds.

### 2.5. Macrocycles [15]pyN_5_ and [16]pyN_5_ Interact with the Catalytic Zinc and its Coordinating Amino Acids

In silico docking studies were performed to better understand the molecular mechanism of interactions of [15]pyN_5_ and [16]pyN_5_ with MMP-2 and MMP-9. The reference inhibitor ARP-100 was also docked in the same conditions.

**Figure 7:**
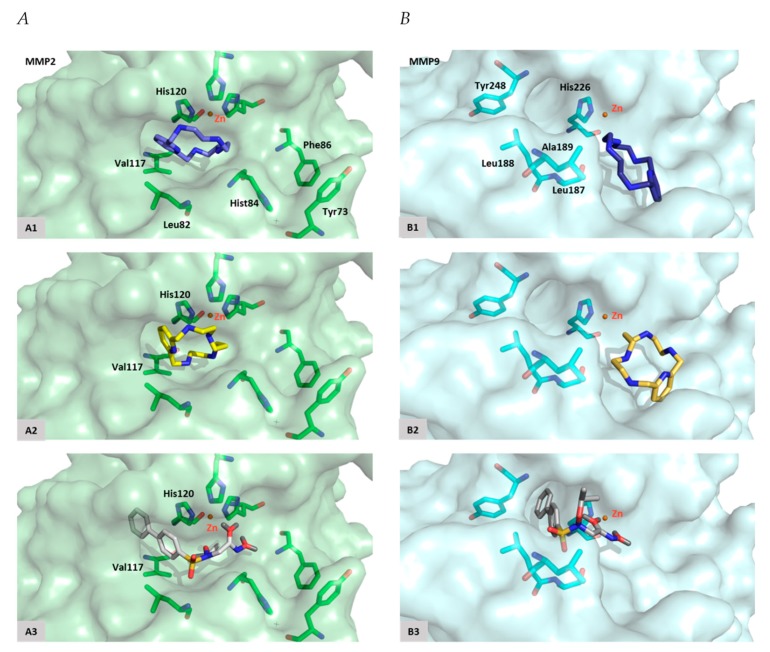
Docking poses. (**A**) MMP-2 active site with [15]pyN_5_ (**A1**), [16]pyN_5_ (**A2**) and ARP-100 (**A3**); (**B**) MMP-9 active site with [15]pyN_5_ (**B1**), [16]pyN_5_ (**B2**) and ARP-100 (**B3**).

Regarding MMP-2, docking studies reveal that [15]pyN_5_ and [16]pyN_5_ mainly occupied the pocket S1′ (Figure 7). One of the nitrogen atoms from the compounds interact with the catalytic Zn^2+^ and possesses similar distances when compared with ARP-100 (2.4 vs. 2.1–2.6 Å). An interaction between the phenyl group of [15]pyN_5_ and [16]pyN_5_ and the imidazole group of His120 (from MMP-2) was found in a distance similar to the reference inhibitor ([15]pyN_5_ and [16]pyN_5_ ~ 3.0–3.6/3.8–3.8 Å vs. ARP-100 ~ 3.0–3.5 Å- Table 1). This interaction plays an important role in stabilization of the molecules into the catalytic site. Other favorable hydrophobic interactions are observed with Leu82, Val117 and His84 (Table 1).

Finally, some MMP-2 known inhibitors were also docked. It was shown, for MMP-2 structure, that the known inhibitors occupy mainly the S1′ pocket, interacting with Zn^2+^ ion (~ 2.0 Å - Table S3). According to the results, the known inhibitors interact with His120 (π-π interaction), Leu82 (H-acceptor) and Val117 similarly to [15]pyN_5_ and [16]pyN_5_ compounds. Besides that, some important interactions are observed with S3 pocket amino acids (Tyr73, His84 and Phe86) of MMP-2 (Table S3).

Concerning MMP-9, [15]pyN_5_ and [16]pyN_5_ present an interesting pose, occupying mainly the opposite region to S1′ (Figure 7). The Zn^2+^ distance varies between 2.6 Å for ARP-100 and 4.0–4.4 Å to [15]pyN_5_ and [16]pyN_5_ (Table 1). As depicted in Figure 7 the phenyl groups of ARP-100 occupy S1′pocket: a π-π interaction appears between these groups and the imidazole group of His226. Tyr248, an amino acid located on the opposite region of the S1′pocket, also interacts with ARP-100. It was also observed an H-bond formation with Leu188 and a strong interaction with Ala189 and Leu187. These interactions revealed to be similar to the binding interaction observed for [15]pyN_5_ and [16]pyN_5_. Overall, relevant interactions, with similar distances, were observed between the molecules [15]pyN_5_, [16]pyN_5_ and ARP-100 and the important amino acids of the binding pocket of MMP-9.

### 2.6. Cell migration is Significantly Impaired by [15]pyN_5_ and [16]pyN_5_

To evaluate the effect of the test compounds in cancer cell migration, scratch and radial migration assays were performed in 2D and 3D cultures, respectively, exposed to [16]pyN_5_, [15]pyN_5_ and ARP-100.

**Figure 8:**
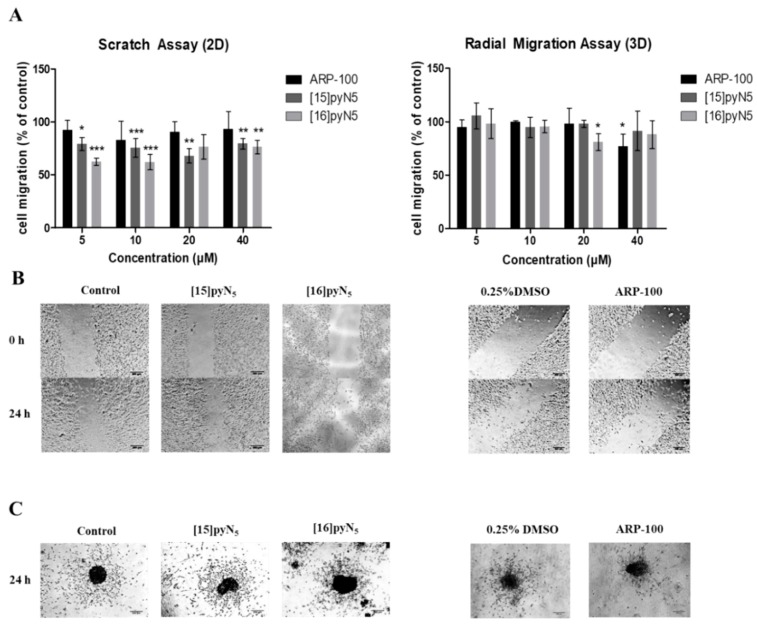
Effect of [15]pyN_5_, [16]pyN_5_ and ARP-100 in cell migration in 2D (scratch) and 3D (radial migration) models. (**A**) Quantification of migrated cells after 24h in 2D and 3D models of MDA-MB-231 cells. Data expressed as percentage (mean ± SD, *n* = 3–4) relative to controls (non-treated condition for [15]pyN_5_ and [16]pyN_5_ and 0.25% DMSO for ARP-100); (**B**) representative images of scratch assays after 0 and 24 h upon incubation with 20 μΜ of each compound and respective controls, scale bars = 200 μm; (**C**) representative images of radial migration after 24 h upon incubation with 20 μΜ of each compound and respective controls. Magnification 4×, scale bars = 200 μm. Statistical significance is represented as * *p* < 0.05, ** *p* < 0.01, *** *p* < 0.001.

In monolayer cultures, scratch assays were performed in serum-free conditions, so that the closure of the scratch would result of cell migration instead of proliferation. Herein, a significant inhibitory effect was observed for both macrocyclic compounds, when comparing to the non-treated control condition. For concentrations as low as 5 μM (non-cytotoxic) [16]pyN_5_ and [15]pyN_5_ reduced cell migration 38 ± 2% (*p* < 0.001) and 21 ± 4% (*p* < 0.05), respectively (Figure 8A,B). ARP-100, on the other hand, when compared to the corresponding control (0.25% DMSO), did not induce a significant inhibitory effect on cell migration at any concentration tested (Figure 8A,B).

For the radial migration assays, 10% FBS supplemented medium was adopted, as serum-free medium alone hampered cell migration ability (data not shown). As depicted in Figure 8A,C, both [15]pyN_5_ and [16]pyN_5_ macrocycles were not particularly effective in decreasing spheroid migration. In fact, only [16]pyN_5_ at a concentration of 20 μM was able to show a significant effect with cell migration decreasing 20 ± 4% (*p* < 0.05). Similarly, treatment with ARP-100 resulted in a significant cell migration decrease of 23 ± 3% relative to control (0.25% DMSO) only at 40 μM (*p* < 0.05).

### 2.7. Breast Cancer Cell Invasion Ability is Maintained upon Exposure to [15]pyN_5_ and [16]pyN_5_

The invasion ability of MDA-MB-231 2D cultures in the presence of the compounds was performed through a chemotaxis assay, using transwell inserts coated with Matrigel, which mimics the presence of extracellular matrix that cells have to degrade in order to invade surrounding tissues. In spheroid cultures, radial invasion assays were adopted.

**Figure 9:**
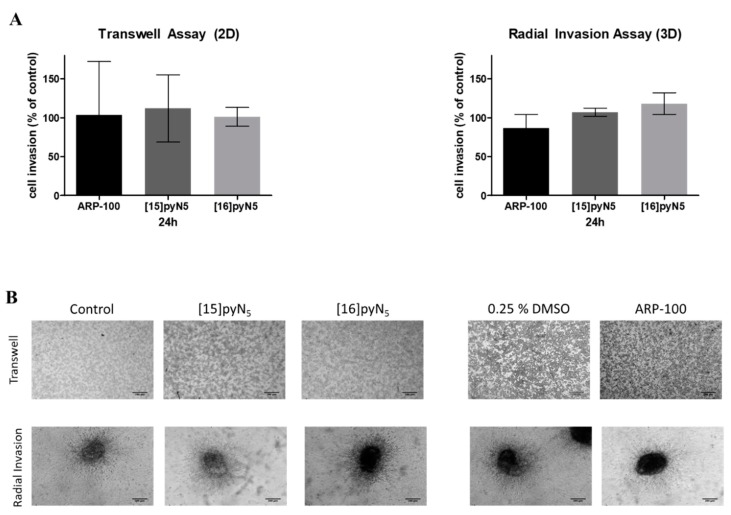
Effect of [15]pyN_5_, [16]pyN_5_ and ARP-100 in cell invasion in 2D (Matrigel^TM^ coated transwells) and 3D (radial invasion) models. (**A**) Quantification of MDA-MB-231 cell invasion after 24 h in 2D and 3D cultures. Data expressed as percentage (mean ± SD, *n* = 3–4) relative to controls (non-treated condition for [15]pyN_5_ and [16]pyN_5_ and 0.25% DMSO for ARP-100); (**B**) representative images of transwell assays and of radial invasion assays after 24 h upon incubation with 20 μΜ of each compound and respective controls. Magnification 4×, scale bars = 200 μm.

Herein, the macrocyclic compounds and ARP-100 showed no significant inhibitory effect on cancer cell invasion (Figure 9). Nevertheless, spheroids treated with ARP-100 revealed a slight decrease of 14% on its invasion ability at 24 h when compared to non-treated condition (n.s.) (Figure 9). This decrease was already observed at 16 h (data not shown).

## 3. Discussion and Conclusions

In this study a comprehensive multidisciplinary approach combining physiologically relevant in vitro models and docking studies was used for evaluating the MMP-2 and 9 inhibitory potential of two pyridine containing-pentaaza macrocycle compounds for breast cancer therapy.

MDA-MB-231 have been described as highly proliferative triple-negative breast cancer cells belonging to the Basal-like tumors’ type [39]. It has also been described as expressing a claudin-low phenotype [40]. Indeed, one of the main differences between basal-like cell lines and the primary tumor identified as claudin-low is the proliferation gene cluster, which can be regarded a result of the selection imposed on cell lines under standard in vitro conditions. Moreover, despite several similarities among the basal-like and claudin-low subtypes, it has been even proposed that these two subtypes might not display the same sensitivity to anthracycline/taxane-based chemotherapy treatments [40]. As such, 3D models may provide solid insights that allow for a more competent cell-type/subtype characterization, by revealing information that can be masked under non-physiological culture conditions [28,29,41]. In this work, monolayer and spheroid cultures presented relevant differences, particularly in terms of ECM presence and cellular organization inducing different proliferation profiles and gelatinase activity, which translated into different sensitivity to the compounds tested as prospective anti-cancer agents.

Results from cell viability assays showed that MDA-MB-231 in monolayer were significantly more affected by both macrocyclic compounds and ARP-100 when compared to the cells aggregated in spheroids, in which cell viability was consistently higher than ~75%, even for the highest compound concentrations. This 3D-higher cell resistant profile has been proposed to be related to features usually lacking in standard 2D conditions, namely quiescence, ECM-mediated signaling pathways (e.g., apoptotic pathways) [42,43] and even hypoxia-mediated P-glycoprotein up-expression [44]. Thus, similarly to the gradient of nutrients and oxygen, drug penetration is also somewhat hindered in spheroids, once again mimicking the hindered diffusion in solid tumor [45,46]. In fact, proliferation marker Ki-67 has been used as a prognostic marker for breast cancer, with reported values under 50% Ki-67^+^ cells in most in vivo tumors, clearly differing from the 100% percentage of Ki-67^+^ cells present in breast cancer cells cultured in monolayer [39]. Obtaining an in vivo-like proliferation profile is thus vital for a more realistic screening of potential anti-cancer agents, as well as to better understand breast cancer cell heterogeneity and provide a more solid classification.

Besides the differential cell sensitivity and proliferative indices, a culture-dependent gelatinase secretion profile was also observed amongst MDA-MB-231 cells in both culture conditions, with higher expression of MMP-9 and MMP-2 in spheroids and monolayer cultures, respectively. Previous reports stated that MDA-MB-231 cells cultured in a fibronectin coated surface induced both higher expression and activity of MMP-9 [47]. The more complex cell-cell and cell-ECM interactions present in the spheroids might have a role in this secretome change, as cell adhesion molecules such as integrins have been described to modulate MMPs expression [47].

On the other hand, zymography assays performed in this study showed that ARP-100 presents selectivity to MMP-2, with an overall higher inhibition of this gelatinase in both 2D and 3D when compared to MMP-9. This was expected, as this molecule contains an alkyl substituent at the carbon atom adjacent to the hydroxamic acid, that confers lipophilic interactions to the S1 region of the active site, allowing a greater selectivity for MMP-2 in detriment of other MMPs. Notably, both [15]pyN_5_ and [16]pyN_5_ inhibited gelatinases at concentrations lower than that of ARP-100. Data from the pZn^2+^ and species distribution for both compounds support their affinity for Zn^2+^ chelation, which is consistent with their inhibitory effect. Despite its lower interaction towards the S1 pocket due to their structure, both [15]pyN_5_ and [16]pyN_5_ interactions with Zn^2+^ may have been the base for this higher inhibition of gelatinases when compared to ARP-100, as MMPs function relies on its structural Zn^2+^ ion (Figure 6).

Indeed, docking studies confirmed the interaction of the compounds with MMP-2 and MMP-9. The poses’ differences observed between MMP-2 and MMP-9 can be explained considering the shapes and the volumes of S1′ hydrophobic pockets (Figure 7). Although the structures of MMP-2 and MMP-9 are highly similar the residues comprising the S1′ loop share no amino acid sequence similarity and differ in length, justifying the differences observed between the two pockets. [15]pyN_5_ and [16]pyN_5_ in particular, appear to be the compounds with higher affinity to MMP-2, presenting an interesting position in S1′ pocket, namely interacting with His120 (π-π interaction) and a Zinc distance similar to the observed for the reference compound ARP-100 (Table 1). The longer distance, and lower interaction, observed between these molecules and S3′ pocket residues His84 and Tyr73 can nevertheless be overcome with the design of a chain that is able to strongly interact with these residues. Moreover, in order to optimize the interactions between [15]pyN_5_ and [16]pyN_5_ and S1′ pocket of MMP-9, modifications in size of these macrocyclic could be proposed.

Although herein the computational methods show interaction of these py-macrocycles with MMP-2 and 9, due to MMPs highly conserved zinc-dependent catalytic site, [15]pyN_5_ and [16]pyN_5_ probably also inhibit other MMPs. The importance of MMPs for invasion has been described, but its role in migration per se is still being disclosed. Specifically, in scratch assays, inhibition of MMPs through gene silencing antibody neutralization, and pharmacological inhibitors such as batimastat [11,12,13,14] have not been able to completely ablate cell migration. Since in the in vitro scratch assay conditions, MMPs will probably act by cleaving cell-attachment receptors directly, thus modulating cells adhesion during migration [48] and triggering signaling pathways involved in migration and angiogenesis [49]. MMP-1, MMP-9 and MMP-2 were shown to cleave CD44, to cleave CD44 and integrin α_M_β2, and to cleave integrin αVβ3 [34,50], respectively, further promoting migration. Besides, it is noteworthy that MMPs function extends beyond proteolytic functions, as their hemopexin domains have been shown to have important roles in migration [51,52,53]. Indeed, Dufour et al. showed that gelatinase-induced migration did not depend on their proteolytic functions, showing that MMP-9-induced migration was dependent of its hinge and hemopexin domains rather than due its adhesion ability [54]. In this study, MDA-MB-231 cellular migration results also suggest that spheroids are less sensitive than 2D models to the tested compounds. ECM such as Matrigel^TM^_,_ which was present in the 3D culture both embedded in the spheroids and in the coating, has been indicated to influence cell migration upon exposure to chemicals. Millerot-Serrurot et al. [55] described that HT1080 cells in 3D collagen I matrix had no significant migration inhibition, at the same doxorubicin concentrations that induced 70% inhibition in 2D models. Moreover, the GM6001 broad MMPi abrogates ovarian cancer cell monolayers and spheroids migration in collagen I matrix but not in Matrigel^TM^, showing the relevance of ECM elements for migration and invasion [56]. Moreover, as mentioned above, similarly to in vivo, the compounds might not diffuse completely to the more central zone of the spheroid, affecting just the periphery.

Cellular migration and invasion are two landmark events in cancer progression and metastization. Herein, MDA-MB-231 migration was significantly reduced by the tested compounds, whereas the same was not observed in terms of invasion impairment. The reasons for this differential outcome are not clear and future work is needed to unravel the mechanism underlying this effect. However, it should be noted that cancer cell migration and invasion although coordinately regulated are distinct processes [57].

In sum, the new findings from this study strongly demonstrate that [15]pyN_5_ and [16]pyN_5_ are potential inhibitors of MMPs 2 and 9, suggesting its involvement in the observed impaired cell migration of breast cancer cells and therefore reinforcing their potential use as prospective drugs for cancer therapy.

## 4. Methods

### 4.1. Chemicals

Macrocyclic compounds [15]pyN_5_ and [16]pyN_5_ (Figure 1A) were synthesized in house according to previously described methods [20], using a template procedure for the cyclization (Figure 1B). The macrocycles were prepared in good yields and characterized by ^1^H and ^13^C NMR spectroscopy (Tables S1 and S2 and Figures S1 and S2) using a Bruker Avance 400 spectrometer (Bruker BioSpin GmbH, Rheinstetten, Germany) and by Melting points and Elemental analyses using a Leco TruSpec Micro Elemental Analyzer (LECO Corporation, St. Joseph, MI, USA) (see Supplementary information), revealing a very high degree of purity. Aqueous solutions of both compounds were prepared at 2 mM, and its exact concentrations were determined by potentiometric titrations.

The aqueous solutions of [15]pyN_5_ and [16]pyN_5_ used in the studies were prepared by adjusting the pH to 7.4 with a KOH solution. Additionally, a 40 mM stock solution of ARP-100 (Santa Cruz Biotechnology, CA, USA) was prepared in DMSO (Sigma-Aldrich^®^, St. Louis, MO, USA). Final DMSO concentration was kept at 0.25% (*v*/*v*) in cell cultures throughout the experiments.

### 4.2. Cell Culture

Human breast carcinoma cell line MDA-MB-231 was purchased from ATCC (American Type Culture Collection, Manassas, VA, USA). In monolayer cultures, cells were inoculated in Dulbecco’s modified Eagle’s medium (DMEM) (Sigma-Aldrich^®^, St. Louis, MO, USA) supplemented with 10% fetal bovine serum (FBS-Gibco^®^, ThermoFisher Scientific, Waltham, MA, USA).

For spheroid generation, 6-well, 24-wells and 96-wells ultra-low attachment (ULA) flat-bottomed plates (Corning^®^, NY, USA) were inoculated with 250,000 cells/mL in 1500 μL/well, 300 μL/well and 50 μL/well, respectively. Culture medium consisted in DMEM supplemented with 10% FBS and 2% Growth Factor Reduced Matrigel^TM^ (Corning^®^, NY, USA). Cultures were kept under a humidified atmosphere with 5% CO_2_ at 37 °C. Spheroids were then incubated for up to 6 days, with medium replacement every 3 days.

### 4.3. Histology

#### 4.3.1. Haematoxylin and Eosin (H&E) Staining

Cryosections of 5–7 µm, prepared from 3D spheroids (from day 4) ressuspended in Tissue Tek^®^ O.C.T.™ (Sakura, Alphen aan den Rijn, The Netherlands), were fixed with cooled acetone for 10 min. Slides were first stained with Harris’s haematoxylin (Sigma-Aldrich^®^, St. Louis, MO, USA) for 20 min, followed by Eosin Y (Sigma-Aldrich^®^, St. Louis, MO, USA) staining for 2 min. Slides were then submitted to increasing concentrations of ethanol and finally incubated in xylene (EMD Chemicals, Burlington, MA, USA). Images were acquired with an Olympus CK30 inverted microscope using Motic Images Version 2.0 software.

#### 4.3.2. Ki-67 Staining

Spheroid cryosections were fixed with pre-cooled acetone for 30 min at −20 °C, left drying, and then permeabilized with 0.08% Tween 20^®^ for 2 min at room temperature. This was followed by a 30 min blocking with 2% bovine serum albumin (BSA) in PBS. Incubation with primary antibody was performed overnight in a humidified chamber at 4 °C. The primary antibody used was: Ki67 (Rabbit IgG, AB16667) (Abcam, Cambridge, United Kingdom) diluted 1:100 in 1% BSA in PBS. The incubation with the secondary antibody goat anti-rabbit 594 (1:500) (Invitrogen™, Thermo Fisher Scientific, Waltham, MA, USA) was carried out for 1h at room temperature. Sections were mounted using ProLong gold antifade with DAPI (4′,6-diamidino-2-phenylindole, Invitrogen™) and analyzed using an inverted fluorescence microscope (Axiovert 200 M) (Carl Zeiss, Oberkochen, Germany) coupled with a monochrome camera (AxioCam MNC) (Carl Zeiss, Oberkochen, Germany). Sample fluorescence was examined at excitation/emission wavelengths of 590/617 nm (Alexa Fluor 594) and 358/461 (DAPI). Images were collected using AxioVision Rel. 4.7 software.

### 4.4. Cell Viability Assays

The cytotoxicity of [15]pyN_5_, [16]pyN_5_ and ARP-100, herein used as control, was evaluated in MDA-MB-231 cells in both 2D and 3D culture conditions using CellTiter 96^®^ AQueous One Solution Cell Proliferation Assay (MTS) (Promega, Madison, WI, USA). In 2D cultures, 8 × 10^3^ cells were seeded in 96-well plates and maintained for 24 h in complete culture medium. Thereafter, cells were exposed to 100 μL of [15]pyN_5_, [16]pyN_5_ and ARP-100 at final concentrations of 1-100 µM, in serum free DMEM. After 24 h incubation, 20 μL of MTS were added to the wells and left incubating for 1 h [58]. The absorbance was recorded by spectrophotometry at 490 and 690 nm (SPECTROstar Omega) (BMG LABTECH, Ortenberg, Germany). Three independent experiments were performed.

In 3D cultures, the cytotoxicity assays were performed in 4-day spheroid cultures in 96-well ULA plates. As such, spheroids’ culture medium was replaced by 100 µL of DMEM with 1.25% FBS, 2% Matrigel^TM^ and [15]pyN_5_, [16]pyN_5_ and ARP-100, (1–100 µM). After 24 h, spheroids were incubated 1 h with MTS. Spheroids were pelleted and the supernatant read by spectrophotometry at 490 nm and 690 nm. The number of viable cells was assessed by the determination of the difference of absorbance, A_490 nm_–A_690 nm_ and cell viability was expressed as percentage to control. Three independent experiments were performed.

### 4.5. Production of Conditioned Media

For the production of the conditioned medium under 2D conditions (CM2D), 1.5 × 10^6^ MDA-MB-231 cells were seeded in 75 cm^2^ T-flask for 24 h. At this point, 15 mL of serum-free DMEM were added to the cells. 3D conditioned medium (CM3D) was performed at day 4 of culture, in 6-well ULA plates. Spheroids were washed twice with serum-free DMEM, and medium was replaced by 2 mL of serum free DMEM supplemented with 2% Matrigel^TM^. In both systems, medium was conditioned for 24 h, being afterwards harvested and concentrated 100× using 10 kDa centrifugal concentrators (Millipore). Total protein was quantified by spectrophotometry (SPECTROstar Omega) (BMG LABTECH, Ortenberg, Germany) in a low volume microplate (Lvis plate) (BMG LABTECH, Ortenberg, Germany). Total protein concentration was determined through Lambert-Beer equation, with absorbance 280–340 nm and extinction coefficient of bovine serum albumin.

### 4.6. Docking Studies

#### 4.6.1. Protein and Ligand Structures

Initial protein structures of MMP-2 and MMP-9, PDB codes: 3AYU and 4XCT (resolution 2.0 Å and 1.3 Å respectively), were selected and obtained from PDB database. The protein structures were prepared using Molecular Operating Environment (MOE) software version 2018.01 (Montreal, QC, Canada). Water molecules and crystallographic ligands were removed from the original crystallographic protein structures. After removal the protein and all the ligands were protonated for the pH 7.4, using Protonate3D feature implemented in MOE software (some ligands protonation states also adjusted manually). The ligands energies were minimized with Amber2012 force field. The previously prepared ligands were docked into 3AYU and 4XCT protein binding site using GOLD 5.20 (Genetic Optimization for Ligand Docking) software. Default speed settings were accepted and used during the docking simulations. The Genetic Algorithm runs were defined as 500. The 10 best results for each ligand were kept. All the simulations were performed with ChemPLP and GoldScore scoring functions.

To validate the prepared MMP protein structures, a first docking calculation was performed to confirm if the docking protocol followed (protein structure adequacy and all docking parameters) was able to predict ligand poses in agreement with experimental results. In this first docking step, the peptide APP-IP (present in the MMP-2 crystallographic structure, 3AYU) and n73 (N2-[biphenyl-4-yl(dihydroxy)-lambda4-sulfanyl]-N-oxo-N2-(propan-2-yloxy)-D-valinamide, present in the MMP-9 crystallographic structure, 4XCT) were docked on the respective active sites using a rigid receptor methodology. The top solutions (the 10 poses with the highest score, for each ligand) were critically compared to the crystallographic poses validating our protocol.

After the protocol validation, compounds [15]pyN_5_, [16]pyN_5_, ARP-100 and the commercial specific inhibitors 420121-84-2, 582311-81-7, 848773-43-3, 868368-30-3, Prinomastat, Rebimastat, Ro-28-2653, Sb-3CT, Tanomastat and YHJ-132 (MMP-2 specific) [59,60,61] were docked into the respective protein active site. The best 10 poses for each compound were critically evaluated inside the pocket and the most important protein-ligand interactions were assessed. Considering the results of Goldscore and ChemPLP scoring functions it was observed that ChemPLP could be the scoring function that better described the system and the binding mode. Only ChemPLP results were discussed. The results are presented as supplementary information (Table S4).

#### 4.6.2. MMP-2 and 9 Binding Sites Definition

The catalytic zinc defined both MMP-2 (2541 atom in 3AYU) and MMP-9 (2404 atom in 4XCT) binding sites. Besides, in MMP-2 two pockets are important to ligands interactions: S1′ with His120, Leu82 and Val117; and S3 with Tyr73, His84 and Phe86. In MMP-9 the amino acids Leu187, Leu188, Ala189, His226 and Tyr248 shown to be relevant to establish interactions with known ligands.

### 4.7. Modified Zymography Assay

To evaluate MMP-2 and 9 total gelatinase activity, a zymography was performed using the methodology described in [62] with modifications as depicted in Figure 2. CM concentrated samples were resolved by electrophoresis under non-reducing conditions, in a 10% Polyacrylamide gel containing 0.1% SDS and 0.1% (*w*/*v*) gelatin. After electrophoresis, the lanes were individualized and washed with 2.5% Triton X-100 in 3 steps of 20 min, to remove SDS. Gel lanes were incubated for 24 h at 37 °C in Developing Buffer (50 mM Tris Base, 200 mM NaCl, 5 μM ZnCl_2_, 5 mM CaCl_2_.2H_2_O and 0.02% NaN_3_) containing [15]pyN_5_, [16]pyN_5_ and ARP-100 (5–20 µM). Incubation with developing buffer restores MMPs’ protease activity. Therefore, incubations in the absence of compounds were also performed in parallel as control. Finally, gels were stained with 0.1% Coomassie Brilliant Blue R-250 (National Diagnostics, Charlotte, NC, USA). MMP activity was detected as a clear band in the background of uniform staining. Band quantification was performed using ImageJ software (National Institutes of Health, Bethesda, MD, USA).

### 4.8. Migration Assays

#### 4.8.1. Scratch Assay

The effect of pyridine containing macrocycles on cell migration was evaluated through in vitro scratch assay according to previously published procedures [63,64]. Briefly, 2 × 10^5^ MDA-MB-231 cells were seeded in 24-well plates in complete cell culture medium. After 24 h, the medium was removed and a scratch was performed in each well using a 200-µL pipette tip. Cells were then rinsed twice with PBS and kept in serum-free medium containing test compounds [15]pyN_5_, [16]pyN_5_ or ARP-100 in concentrations ranging from 5–40 µM. The scratch was evaluated microscopically (AE 2000 inverted microscope) (Motic, Hong Kong, China), and four images of each scratch were recorded using Moticam 2500 (Motic, Hong Kong, China) at defined time-points: 0, 5, 10 and 24 h. Non-invaded distance was measured using Motic Images PLUS v2.0 software. Three independent experiments were performed.

#### 4.8.2. Radial Migration

Radial migration was performed in 3D cultures according to Vinci et al. [26]. Flat-bottomed, 24-well plates (Sarstedt, NC, USA) were coated with 0.01 mg/mL Poly-D-Lysine (Sigma-Aldrich^®^, St. Louis, MO, USA) in sterile water for 2 h at room temperature, followed by 1:30 diluted Matrigel^TM^ coating for 30 min. Four-day spheroids were transferred to pre-coated plates and maintained in 500 μL of medium containing 10% FBS, 2% Matrigel^TM^ and the compounds, [15]pyN_5_, [16]pyN_5_ and ARP-100, in concentrations ranging from 5–40 µM. Images were captured at 5, 10 and 24 h using AE 2000 inverted microscope (Motic, Hong Kong, China) and the area between cells in the migration front and the perimeter of the spheroid measured. More than 10 spheroids per condition were analyzed.

### 4.9. Invasion Assays

#### 4.9.1. Chemotaxis/Chemoinvasion Assay

For 2D cultures, the screening of the chemotactic invasion of MDA-MB-231 cells was evaluated in 24 well-plates with transwell inserts (Corning^®^, NY, USA) coated with a solution of 0.3 mg/mL Matrigel^TM^ (Corning^®^, NY, USA), which blocked the passage of non-invasive cells. After 1-h incubation at 37 °C, the non-polymerized coating was removed. At this point, 5 × 10^4^ cells were briefly seeded in the upper chamber in serum free medium. Complete culture medium (chemoattractant) was added to the lower chamber. The test compounds were added to both chambers for a 24 h period of incubation at 37 °C in a humidified incubator (5% CO_2_). At this point, non-invading cells were removed from the upper part of the inserts with a cotton swab. Cells that invaded towards the underside of the inserts were fixed with 96% cold ethanol, and then stained in 0.1% crystal violet solution. Representative images of each condition were taken to measure the stained area. Afterwards membranes were carefully removed from the insert and introduced in Eppendorfs. 10% acetic acid in ethanol solution was added to solubilize the crystal violet. Measurements were performed in a SPECTROstar Omega (BMG LABTECH, Ortenberg, Germany) multiplate reader at 595 nm wavelenght.

#### 4.9.2. Radial Invasion

Screening of MDA-MB-231 invasion ability as spheroids was evaluated through a radial invasion assay. 4-day spheroids were transferred to flat-bottom 24-well plates (Sarstedt, NC, USA) pre-coated with 250 µL of 2 mg/mL neutralized rat tail Collagen solution [produced in house according to [65] and left incubating for 30 min. Medium was removed and another 250 µL of collagen were added over the spheroids, fully covering them. Collagen was allowed to polymerize for 30 min, after which 500 µL of media containing the compounds or respective controls was added and the plate and left incubating at 37 °C in a humidified incubator (5% CO_2_). Representative images of the spheroids were taken at 16 and 24 h and the effects of compounds analyzed by measuring the area between cells in the migration front and the perimeter of the spheroid. More than 10 spheroids per condition were analyzed.

### 4.10. Statistical Analysis

Statistical analyses were carried by GraphPad Prism v6.0 software, (La Jolla, CA, USA). ANOVA with Tukey’s multiple comparison post-test was performed for 3 or 4 independent experiments. ANOVA repeated measures tool was used when there were values matching. Results are presented as means ± standard deviation (SD), except where indicated and *p*-values are presented for statistically significant results (* *p* < 0.05, ** *p* < 0.01 and *** *p* < 0.001).

## Figures and Tables

**Table 1:** Docking results: score values and interaction distances (in Å) between [15]pyN_5_, [16]pyN_5_ and ARP-100 with MMP-2 and MMP-9 using ChemPLP Scoring function.

		[15]pyN_5_	[16]pyN_5_	ARP-100
MMP-2	Score	53.1	53.3	93.2
	Zn^2+^	2.1	2.6	2.4
	His120	3.6	3.8	3.5
	Leu82	4.7	4.4	2.9
	Val117	3.8	3.0	3.7
	His84	3.7	3.3	3.4
	Tyr73	9.0	10.0	8.9
	Phe86	8.3	8.8	6.3
MMP-9	Score	34.8	42.0	90.0
	Zn^2+^	4.0	4.4	2.6
	Leu187	3.1	4.0	3.9
	Leu188	6.1	6.4	3.2
	Ala189	3.0	4.0	3.1
	His226	5.2	6.5	3.4
	Try248	9.8	10.8	3.6

## Supplementary Materials

Supplementary materials can be found at https://www.mdpi.com/1422-0067/20/20/5109/s1.

## Abbreviations

3D	three-dimensional
[15]pyN_5_	3,6,9,12,18-pentaazabicyclo[1 2.3.1]octadeca-1(18),14,16-triene
[16]pyN_5_	3,6,10,13,19-pentaazabicyclo[13.3.1]nonadeca-1(19),15,17-triene
AHA	acetohydroxamic acid
ARP-100	2-[([1,1′-biphenyl]-4-ylsulfonyl)(1-methylethoxy)amino]-N-hydroxyacetamide
CM2D	Conditioned medium from 2D models
CM3D	Conditioned medium from 3D models
DMEM	Dulbecco modified eagle modified medium
DMSO	dimethylsulfoxide
EDTA	ethylenediaminetetraacetic acid
ECM	extracellular matrix
EMT	epithelial to mesenchymal transition
FBS	fetal bovine serum
H&E	hematoxylin and Eosin
MMPi	MMP inhibitor(s)
MMPs	matrix metalloproteinases
MSS	musculoskeletal syndrome
MTS	tetrazolium salt 3-(4,5-dimethylthiazol-2-yl)-5-(3-carboxymethoxyphenyl)-2-(4- sulfophenyl)-2H-tetrazolium
PAGE	polyacrylamide gel electrophoresis
Py-macrocyles	pyridine containing macrocycles
SDS	sodium dodecyl sulfate
TIMPs	tissue inhibitors of matrix metalloproteinase
ULA	ultra low attachment
