# Supplementary material for: Pyridine-Containing Macrocycles Display MMP-2/9 Inhibitory Activity and Distinct Effects on Migration and Invasion of 2D and 3D Breast Cancer Models
Source: Int J Mol Sci. 2019 Oct 15;20(20):5109. doi: 10.3390/ijms20205109 (PMC6829403; doi:10.3390/ijms20205109)
Supplement: Supplementary file 1 [file ijms-20-05109-s001.pdf]

SUPPLEMENTARY MATERIALS

Characterization of the macrocycles [15]pyN$_5$ and [16]pyN$_5$

The characterization of the compounds was performed by the Melting points (Mp), by Elemental analyses and by ^{1}H and ^{13}C NMR spectroscopy.

The ^{1}H (400.13 MHz) and ^{13}C NMR (100.62 MHz) spectra were recorded on a Bruker Avance-400 spectrometer at 294 K probe temperature. Chemical shifts (δ) were given in ppm and coupling constants (*J*) in Hz. The NMR spectra were performed in $CDCl_3$ (δ ppm ^{1}H: 7.26; 13C: 77.16) or in D_2O. The reference used for the ^{1}H NMR measurements in D_2O was 3-(trimethylsilyl)propionic acid-d_4-sodium salt (DSS) and in $CDCl_3$ the solvent itself (at 7.26 ppm). For ^{13}C NMR spectra 1,4-dioxane (δ ppm: ^{1}H: 3.75; ^{13}C: 67.20) was used as internal reference.

Characterization of the macrocycle [15]pyN$_5$

Melting points and Elemental analyses

Mp 280-2 °C (decomp.). Elemental analyses - Found: C, 37.03; H, 7.16; N, 16.56. Calc. for $C_{13}H_{23}N_5{\cdot}4HCl{\cdot}1.5H_2O$: C, 36.98, H, 7.16, N, 16.59%.

^{1}H and ^{13}C NMR spectroscopy

At pD value 5.10 the ^{1}H NMR spectrum presents six resolved proton resonances (Supplementary Fig. 1A) and ^{13}C NMR spectrum exhibits seven signals (Supplementary Fig. 1B).

The two resonances at low field were assigned to the protons of the pyridine ring, the triplet H_a and the doublet H_b. At high field, [15]pyN$_5$ exhibits four resonances, the singlets at 4.57 and 3.21 ppm assigned to H_c and H_f protons and the triplets at 3.51 and 3.41 ppm to H_d and H_e protons (Supplementary Table 1).

Supplementary Table 1 – Assignment of ^{1}H and ^{13}C NMR data for [15]pyN$_5$ in D_2O at pD 5.10

C/H labels	^{1}H δ (ppm)	*J* (Hz)	^{13}C δ (ppm)
a	7.99 (1 H, t)	8	140.49
b	7.53 (2 H, d)	8	124.50
c	4.57 (4 H, s)	–	50.66
d	3.51 (4 H, t)	6	44.04
e	3.41 (4 H, t)	6	43.56
f	3.21 (4 H, s)	–	44.09
g		–	150.94

A

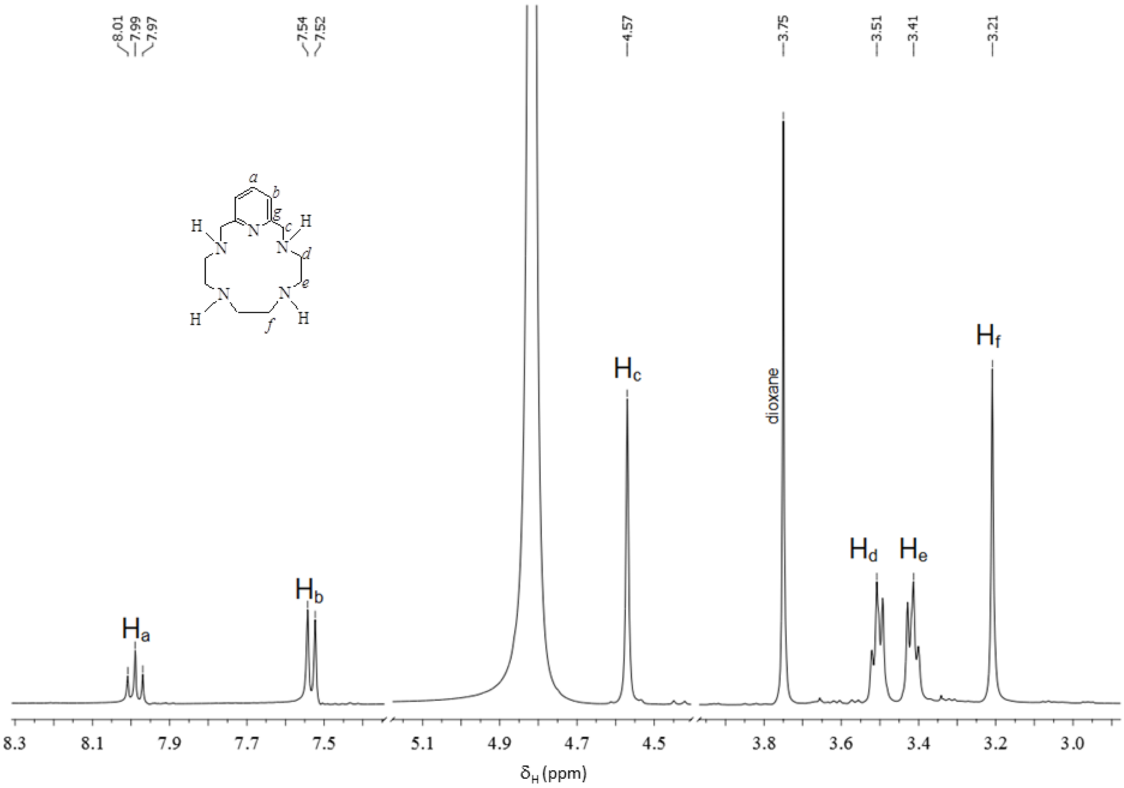

B

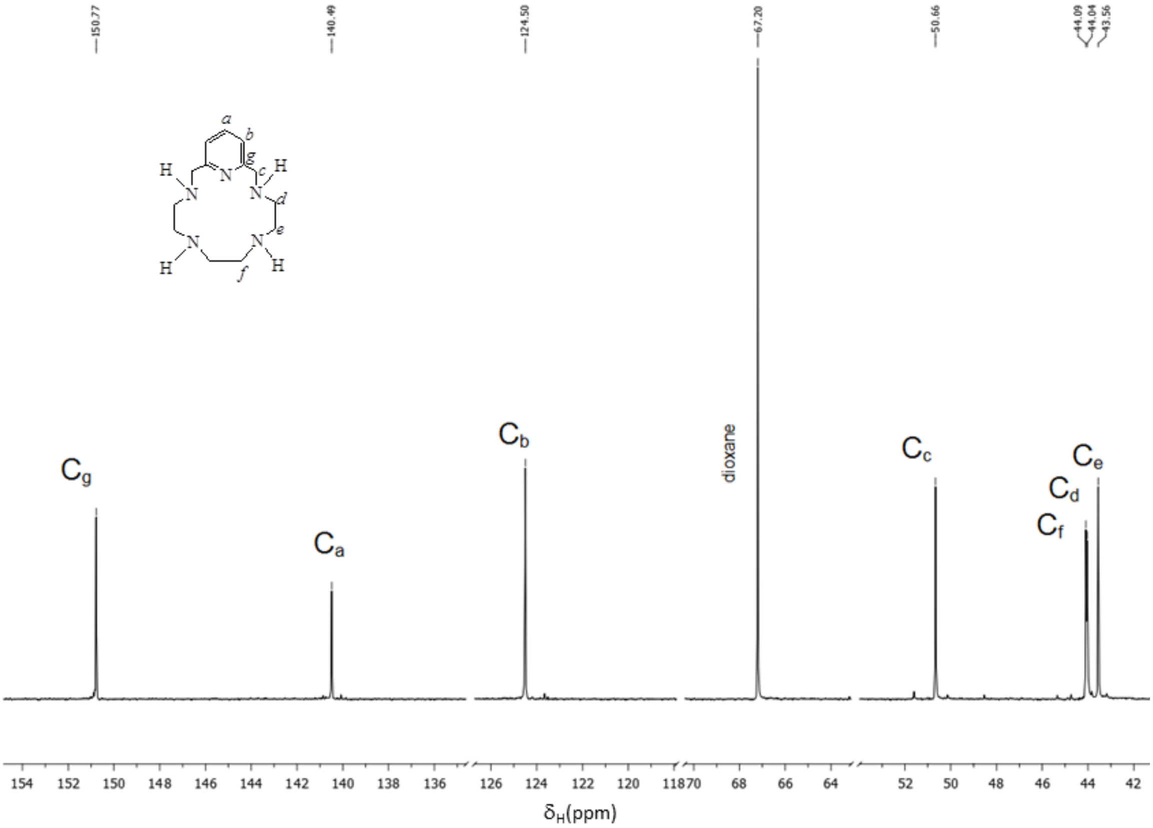

Supplementary Fig. 1 – A) ^{1}H NMR spectrum for [15]pyN_5 in D_2O at pD 5.10. **B)** ^{13}C NMR spectrum for [15]pyN_5 in D_2O at pD 5.10.

Characterization of the macrocycle [16]pyN₅

Melting points and Elemental analyses

Mp 280-2 °C (decomp.). Elemental analyses - Found: C, 37.59; H, 7.80; N, 15.37. Calc. for $C_{14}H_{25}N_5 \cdot 4HCl \cdot 2H_2O$: C, 37.80, H, 7.50, N, 15.70%.

^1H and ^{13}C NMR spectroscopy

At pD value of 2.55 the ^1H NMR spectrum presents seven resonances (Supplementary Fig. 2A). The two resonances at low field were assigned to the protons of the pyridine ring, the triplet H_a and the doublet H_b. At high field the spectrum of [16]pyN$_5$ shows one singlet at 4.63 ppm assigned to H_c protons, three triplets corresponding to H_d, H_e and H_f protons at 3.65, 3.71 and 3.37 ppm and one quintuplet assigned to H_g protons.

^{13}C NMR spectrum exhibits eight signals (Supplementary Fig. 2B). At low field, the spectrum exhibits the three carbons of the pyridine ring C_g, C_a and C_b at δ (ppm) 150.68, 140.44 and 124.50. The two last ones correlate with H_a and H_b resonances at 8.00 and 7.56 ppm. At high field five carbons appear C_c, C_f, C_d, C_e and C_g at δ (ppm) 51.35, 44.14, 43.20, 42.25 and 21.30 and the corresponding ^1H resonances are the singlet H_c at 4.63, three triplets H_f, H_d and H_e at 3.37, 3.65 and 3.71 ppm and finally the quintuplet H_g at 2.21 ppm (Supplementary Table 2).

Supplementary Table 2 - Assignment of ^1H and ^{13}C NMR data for [16]pyN$_5$ in D_2O at pD 2.55

C/H labels	^1H δ (ppm)	*J* (Hz)	^{13}C δ (ppm)
a	8.00 (1 H, t)	8	140.44
b	7.56 (2 H, d)	8	124.50
c	4.63 (4 H, s)	–	51.35
d	3.65 (4 H, t)	6	43.20
e	3.71 (4 H, t)	6	42.25
f	3.37 (4 H, t)	6	44.14
g	2.21 (2 H, q)	7.2	21.30
h	–	–	150.68

A

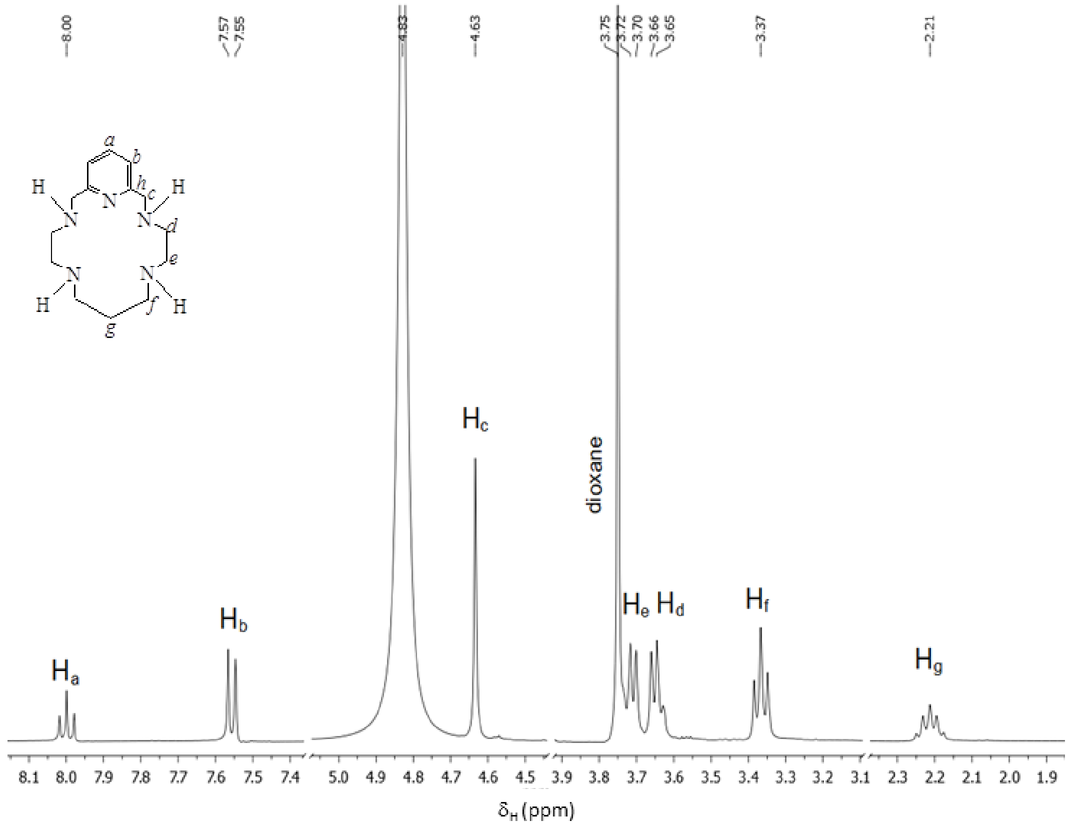

B

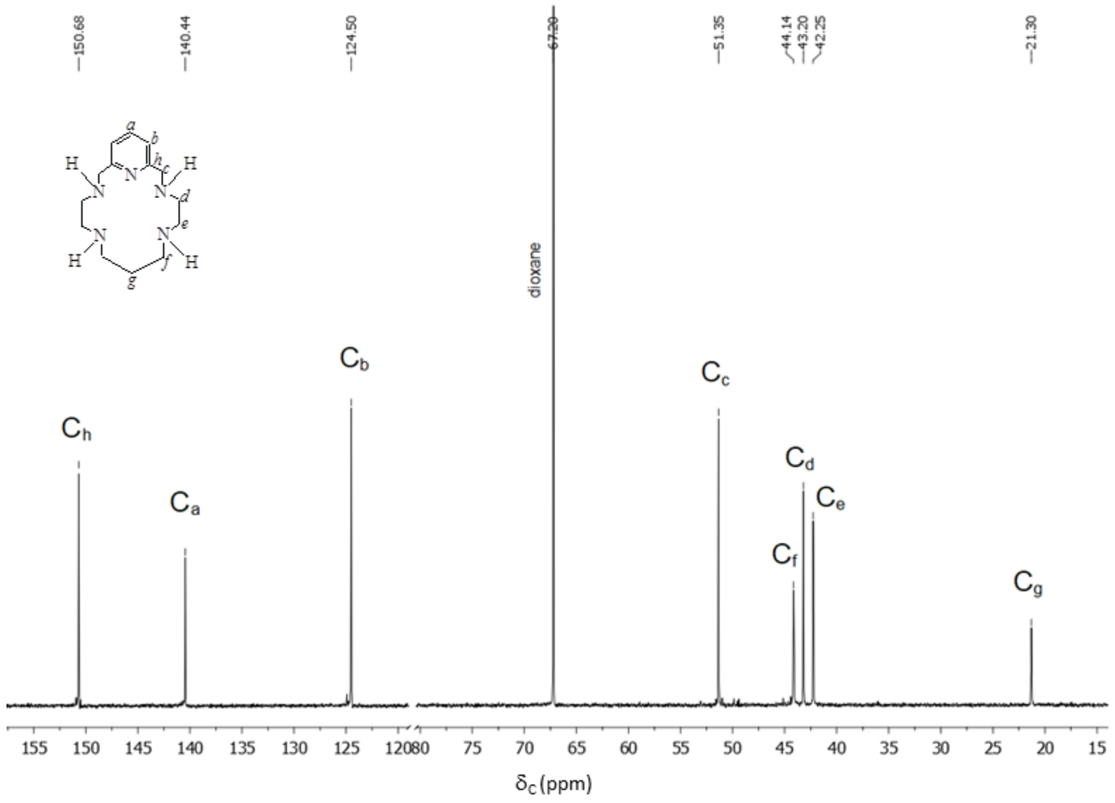

Supplementary Fig. 2 – A) ^{1}H NMR spectrum for [16]pyN_5 in D_2O at pD 2.55; **B)** ^{13}C NMR spectrum for [16]pyN_5 in D_2O at pD 2.55.

Supplementary Table 3 – Collective docking results for known MMP-2 inhibitors (interactions and distance in *Å*) according to Goldscore and ChemPLP Scoring functions.

MMP-2		Score	Zn^{2+} distance	His120	Leu 82	others
				S1'	S1'	
**420121-84-2**	GOLDSCORE	82.28	Coordination 2.34	π-π 3.53		Tyr145; Ala 83; Thr143
	CHEMPLP	96.57	Coordination 2.57		H-acceptor 3.01	Tyr142 Tyr143
**582311-81-7**	GOLDSCORE	93.63				Ala83; Ile141; Tyr142
	CHEMPLP	113.62		H-donor 2.85		Pro140
**848773-43-3**	GOLDSCORE	69.83	Coordination 2.19		H-acceptor 2.89	Tyr142; His130; Ala83
	CHEMPLP	84.51	Coordination 2.54		H-acceptor 2.86	Tyr142 Thr143
**868368-30-3**	GOLDSCORE	82.70	Coordination 1.61	π-π 3.71		Thr143; Ala83
	CHEMPLP	105.56	Coordination 1.99	π-π 3.88		Ala83; Leu137
**Prinomastat**	GOLDSCORE	67.38		π-π 3.63		Thr143; Pro140; Ala 139
	CHEMPLP	76.72	Coordination 2.44		H-acceptor 2.75	Ala 139; His 130; Leu 81
**Rebimastat**	GOLDSCORE	74.23	Coordination 1.85 Coordination 2.45		H-acceptor 2.61	Ala121; Gly80; Ala83
	CHEMPLP	90.06	Coordination 2.60		H- acceptor 3.09	Ala 85; Gly80
**Ro-28-2653**	GOLDSCORE	60.88	Coordination 2.08	π-π 3.24	H-acceptor 3.63	Thr143; Leu81; Gly80
	CHEMPLP	88.79	Coordination 2.58	π-π 3.60		
**Sb-3CT**	GOLDSCORE					
	CHEMPLP	72.29	Coordination 2.61	π-π 3.63	H-acceptor 3.84	Gly80;Leu81
**Tanomastat**	GOLDSCORE	85.69	Coordination 1.94; 2.95	π-π 3.84	H-acceptor 2.86	Ala83; Tyr142; his124
	CHEMPLP	114.17	Coordination 2.35, 2.43		H-acceptor 2.88	Ala83; His124
**YHJ-132**	GOLDSCORE	83.47	Coordination 1.55			Thr143; Pro140; ile141
	CHEMPLP	114.30	Coordination 2.38	π-π 3.95	H-acceptor 2.94	Ala83; Ala 87

Supplementary Table 4- Collective score values, interactions and distance (in *Å*) values between aminoacids of protein and the molecules for ARP-100, [15]pyN$_5$ and [16]pyN$_5$ according Goldscore and ChemPLP Scoring functions.

**MMP-2**		**Score**	**Zn^{2+} distance**	His120	Leu 82	Val117	**Others**
				**S1'**	**S1'**	**S1'**	**S3**
**ARP-100**	GOLDSCORE	37.43	Coordination 2.63	π-π 3.0	H-acceptor 2.5	H-acceptor 3.60	His84 (4.4); Tyr73 (10.4); Phe86(8.7)
	CHEMPLP	93.15	Coordination 2.42	π-π 3.5	H-acceptor 2.9	H-acceptor 3.7	His84 (3.4); Tyr73 (8.9); Phe86(6.3)
**15pyN5**	GOLDSCORE	40.34	Coordination 2.3	π-π 3.0	H-acceptor 5.7	H-acceptor 5.7	His84 (4.4); Tyr73 (9.4); Phe86(8.4)
	CHEMPLP	53.13	Coordination 2.1	π-π 4.2	H-acceptor 4.7	H-acceptor 3.8	His84 (3.7); Tyr73 (9.0); Phe86(8.3)
**16pyN5**	GOLDSCORE	40.86	Coordination 2.4	π-π 3.8	H-acceptor 3.9	H-acceptor 4.8	His84 (3.7); Tyr73 (9.7); Phe86(9.2)
	CHEMPLP	53.26	Coordination 2.6	π-π 4.2	H-acceptor 3.6	H-acceptor 3.3	His84 (3.3 H-acceptor); Tyr73 (10.0); Phe86(8.8)
**MMP-9**		**Score**	**Zn^{2+} distance**	**Leu187**	**Leu188**	**Ala189**	**Others**
							S1'
**ARP-100**	GOLDSCORE	76.54	Coordination 2.1	H-acceptor 3.1	H-acceptor 3.3/2.7	H-acceptor 3.2/2.8	His226 (3.0); Tyr248 (4.4)
	CHEMPLP	90.01	Coordination 2.6	H-acceptor 3.9	H-acceptor 3.2/2.7	H-acceptor 3.1	His226 (3.4); Tyr248 (3.6)
**15pyN5**	GOLDSCORE	44.80	Coordination 3.6	Receptor exposure 3.8	Receptor exposure 7.0	Receptor exposure 3.9	His226 (6.5); Tyr248 (9.6); Ala191 (2.8, H-acceptor)
	CHEMPLP	34.82	Coordination 4.0	Receptor exposure 3.1	Receptor exposure 6.1	Receptor exposure 3.5	His226 (5.2); Tyr248 (9.8); Ala191 (3.0, H-acceptor)
**16pyN5**	GOLDSCORE	39.99	Coordination 2.3	Receptor exposure 5.0	Receptor exposure 5.9	Receptor exposure 2.8	His226 (H-donor, 3.3); Tyr248 (5.8)
	CHEMPLP	41.97	Coordination 4.4	Receptor exposure 4.0	Receptor exposure 6.4	Receptor exposure 4.0	His226 (6.5); Tyr248 (10.8)

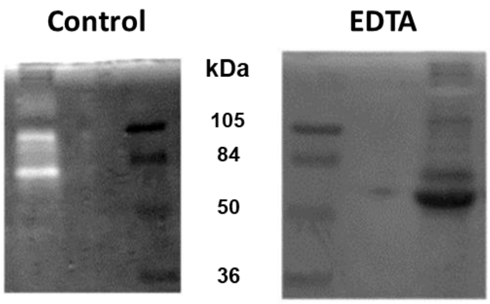

Supplementary Fig. 3 - Zymography gels (CM2D) incubated with and without 7.8 mM EDTA in the developing buffer.
